# Proteomic approaches for characterizing renal cell carcinoma

**DOI:** 10.1186/s12014-020-09291-w

**Published:** 2020-07-29

**Authors:** David J. Clark, Hui Zhang

**Affiliations:** Department of Pathology, The Johns Hopkins University, Baltimore, MD 21231 USA

**Keywords:** Renal cell carcinoma, Proteomics, Protein characterization, Tissue profiling, Serum/plasma profiling, Urine profiling

## Abstract

Renal cell carcinoma is among the top 15 most commonly diagnosed cancers worldwide, comprising multiple sub-histologies with distinct genomic, proteomic, and clinicopathological features. Proteomic methodologies enable the detection and quantitation of protein profiles associated with the disease state and have been explored to delineate the dysregulated cellular processes associated with renal cell carcinoma. In this review we highlight the reports that employed proteomic technologies to characterize tissue, blood, and urine samples obtained from renal cell carcinoma patients. We describe the proteomic approaches utilized and relate the results of studies in the larger context of renal cell carcinoma biology. Moreover, we discuss some unmet clinical needs and how emerging proteomic approaches can seek to address them. There has been significant progress to characterize the molecular features of renal cell carcinoma; however, despite the large-scale studies that have characterized the genomic and transcriptomic profiles, curative treatments are still elusive. Proteomics facilitates a direct evaluation of the functional modules that drive pathobiology, and the resulting protein profiles would have applications in diagnostics, patient stratification, and identification of novel therapeutic interventions.

## Background

Renal cell carcinoma (RCC) is among the top 15 most commonly diagnosed cancers in men and women, with an estimated 73,000 newly diagnosed cases in the United States and 403,000 newly diagnosed cases worldwide [1, 2]. RCC is a heterogeneous disease comprised of three major histopathological subtypes: clear cell renal cell carcinoma (ccRCC), papillary renal cell carcinoma (pRCC), and chromophobe renal cell carcinoma (chRCC); in addition to more rare and benign subtypes that include collecting duct RCC, papillary adenoma, hybrid oncocytic chromophobe, multilocular cystic clear cell carcinoma and oncocytomas [3, 4]. The predominant histology of RCC is ccRCC accounting for ~ 75% of cases, followed by pRCC, which is further divided into two distinct subtypes accounting for 15% of cases, and chRCC which accounts for ~ 5% of cases [5]. To understand the underlying molecular alterations that drive RCC oncogenesis, The Cancer Genome Atlas (TCGA) has performed extensive genomic, epigenomic and transcriptomic profiling of ccRCC, pRCC, and chRCC [6–8]. Unique to ccRCC was the dysregulation of chromosome 3p and associated genes, including the ubiquitous loss of the hypoxic signaling regulator *VHL* and the genes *BAP1*, *PBRM1*, *SETD2*, which have been shown to follow loss of *VHL* and are linked to aggressive disease [9, 10]. Type I pRCC was observed to have a high frequency of chromosome 7, 16, and 17 gain and genomic aberrations involving *MET*, while Type 2 pRCC showed a strong association with *CDKN2A* dysregulation [8]. Genomic alterations in chRCC were quite distinct relative to ccRCC, pRCC, and even other cancer types, observing substantial chromosome copy-number loss, with the majority of tumors displaying loss of chromosomes 1, 2, 6, 10, 13, and 17 [6]. Subsequent pan-renal studies have attempted to delineate the unique and shared features of each histology [11, 12], revealing a high degree of molecular heterogeneity across RCC tumors that could be used to identify up-regulated cellular pathways, immune-related signatures, and patient survival with respect to each histology. Moreover, this molecular information, when combined with histopathological examination, has also provided insight into the nephron cell types from which RCC originates, revealing ccRCC and pRCC arise from proximal tubule epithelial, while chRCC is associated with the distal tubule epithelium [13]. Overall, the genomic and transcriptomic profiling of RCC has provided unique insight into the molecular basis of renal oncogenesis that is complementary to histopathological examination.

Genomic profiling techniques, such as next generation sequencing (NGS), can identify the altered DNA sequences resulting from somatic mutations that are associated with carcinogenesis, as well as serve as a potential screening tool for early diagnosis [14]. However, sequencing DNA and cataloging the altered base-pair profiles provides little information related to the functional consequences of a mutation, and some have questioned the utility genomic-based approaches for identifying actionable targets for therapeutic intervention [15]. Similarly, although transcriptomic analyses offer a high-throughput methodology for assessing gene expression via the measurement of protein-coding mRNA transcript abundance, transcriptomic profiling cannot fully elucidate the functional modules that regulate cellular processes. Mounting evidence has indicated that paired mRNA transcript and protein expression is not robustly correlated in normal tissues or in tumors [16–20], highlighting the rationale for prioritizing protein measurements for an accurate representation of biological systems. Furthermore, mRNA transcript abundance offers minimal insight into post-translational modifications (PTMs) of proteins, such as phosphorylation, glycosylation, or ubiquitination, which serve as additional layers of gene regulation by impacting protein function, stability, protein–protein interactions, and cellular location [21]. Finally, considering that the majority of drug targets are proteins and protein-based analysis is the most common technique utilized in the clinical setting [22, 23], delineating target proteins of interest have direct translational applications. Seeking to address the shortcomings of genomic and transcriptomic profiling, proteomic technologies offer a comprehensive methodology for determining differential global protein and PTM abundance, facilitating a direct analysis of the functionally active molecules and cellular mechanisms dysregulated in the disease state.

**Fig. 1:**
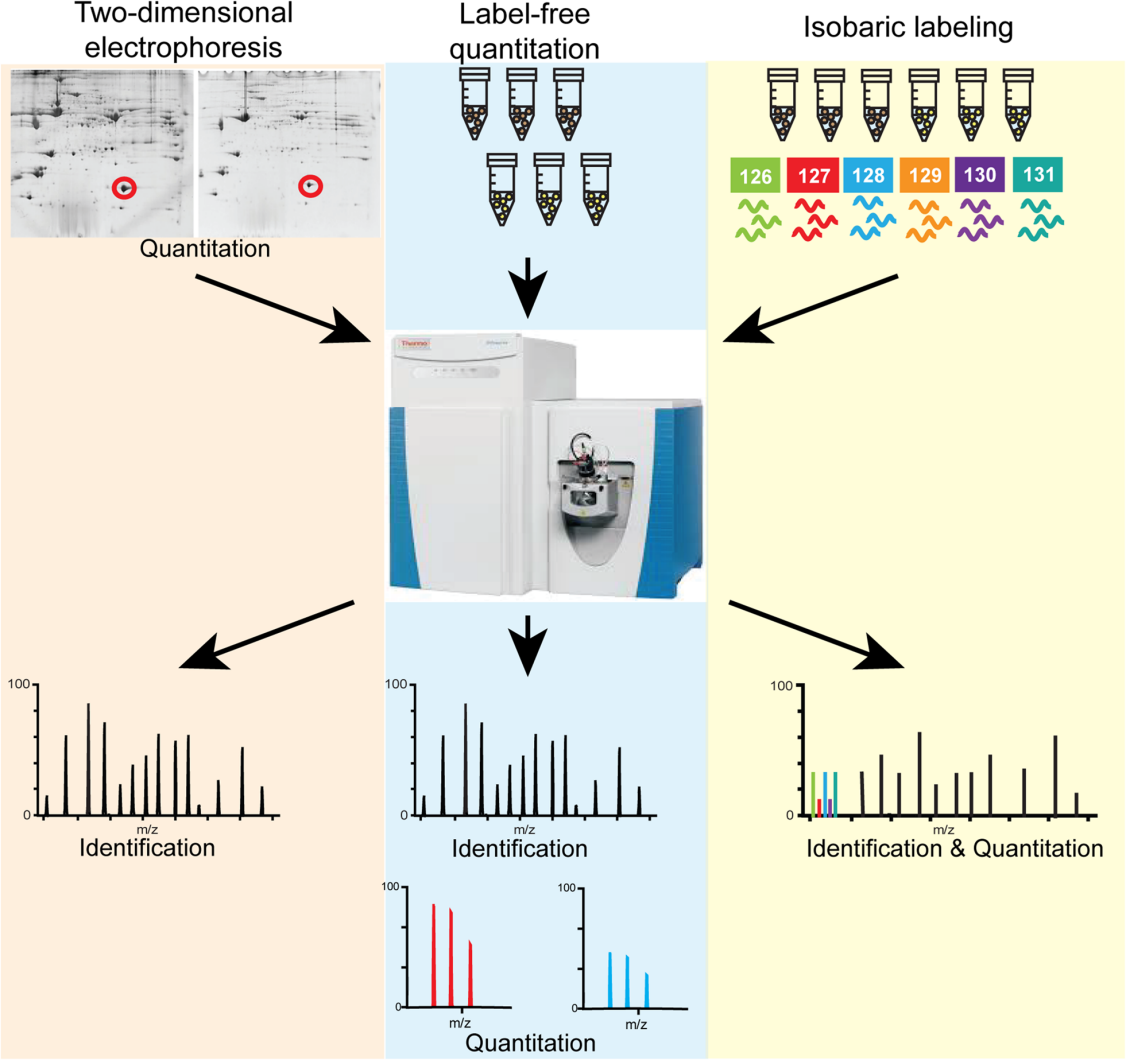
Established proteomic approaches used for investigation of RCC biological samples. Comparative two-dimension electrophoresis (left) assesses protein abundance differences based on spot intensity (Quantitation), with subsequent mass spectrometry analysis identifying the proteins from the excised spot (Identification). Label-free quantitation (middle) entails mass spectrometry analysis of individual samples, with peptides identified at the MS2 level (Identification), and peptide abundance based on peak intensity determined at the MS1 level (Quantitation). Protein abundance is inferred from peptide abundance measurements. In some label-free quantitation-based experiments, spectral counting is employed, wherein protein abundance is inferred by the number of mass spectrometry spectra generated for each peptide derived from the precursor protein. Isobaric labeling (right) methods involve the labeling of peptides derived from individual samples with mass tags that include reporter ions and mixing of samples prior to mass spectrometry analysis. Peptide Identification and Quantitation information is obtained at the MS2 level in the same spectra. Protein abundance is inferred from peptide abundance measurements

There have been a myriad of studies that have utilized proteomic technologies to explore the protein profiles of tissues and biological fluids in an effort to identify the differentially expressed proteins associated with RCC (Fig. 1). In this review, we summarize the details of these studies, focusing our discussion on the samples analyzed, experimental design, proteomic techniques employed, and the results reported in the context of RCC characterization at the protein level. In addition, our review encompasses several emerging approaches and future directions that can be explored to provide further insight into altered protein profiles associated with RCC.

## Tissue profiling

Analysis of tumor tissues offers the most direct method to identify dysregulated protein expression or protein PTM profiles resulting from aberrant genomic events in RCC. Including additional examination of benign or normal adjacent tissues (NATs) facilitates comparative proteomic profiling to identify differentially expressed proteins, with the end goal of delineating the aberrant cellular processes associated with RCC or elucidating potential disease protein biomarkers (Table 1). As a result of these features, the majority of the studies discussed in this review have examined tumor tissues from RCC patients; with these studies representative of various proteomic technologies, RCC histologies, and disease severity.

**Table 1:** Proteins commonly identified as overexpressed in RCC tissues, plasma, serum, urine, or other biological sources

Protein accession	Gene name	Protein name	Biological source	GO annotation^a^	Citing report
P04075	ALDOA	Fructose-bisphosphate aldolase A	T	Canonical glycolysis; actin filament organization	[20, 42, 49]
P05062	ALDOB	Fructose-bisphosphate aldolase B	T,S	Canonical glycolysis	[42, 80]
P09972	ALDOC	Fructose-bisphosphate aldolase C	T	Canonical glycolysis; epithelial cell differentiation	[20, 40, 42]
P04083	ANXA1	Annexin A1	T,S,U	Actin cytoskeleton organization; adaptive immune response	[20, 80, 93]
P07355	ANXA2	Annexin A2	T,U	Angiogenesis	[20, 26, 32, 93]
Q6P452	ANXA4	Annexin A4	T,O	Negative regulation of coagulation	[20, 26, 29, 37, 40, 42, 103, 118]
P52566	ARHGDIB	Rho GDP-dissociation inhibitor 2	T,U	Cellular response to redox state	[63, 93, 119]
P31146	CORO1A	Coronin-1A	T,U	Actin cytoskeleton organization; calcium ion transport	[20, 37, 42, 119]
P02511	CRYAB	Alpha-crystalline B chain	T	Apoptotic process involved in morphogenesis	[20, 28, 30, 40]
P06733	ENO1	Alpha enolase	T	Canonical glycolysis	[28, 41, 42, 64]
P09104	ENO2	Enolase 2	T,O	Canonical glycolysis	[20, 26, 29, 37, 42, 63, 103]
O15540	FABP7	Fatty acid-binding protein, brain	T	Epithelial cell proliferation; triglyceride catabolic process	[26, 27, 37, 40, 61, 63]
P02751	FN1	Fibronectin	T,P	Angiogenesis; cell adhesion	[42, 76, 78]
P00738	HP	Haptoglobin	T,S,U	Acute inflammatory response	[64, 79, 92, 93]
P11142	HSPA8	Heat shock cognate 71 kDa protein	T,S	ATP metabolic process; regulation of protein complex stability	[42, 63, 79, 80]
P04792	HSPB1	Heat shock protein beta-1	T	Cellular response to vascular endothelial growth factor stimulus	[26, 41, 64]
P00338	LDHA	l-lactate dehydrogenase A chain	T	Glycolytic process; response to hypoxia	[20, 26, 29, 40–42, 118]
P09382	LGALS1	Galectin-1	T,O	Apoptotic process; cellular response to glucose stimulus	[20, 27, 46, 103]
Q92597	NDRG1	Protein NDRG1	T	Cellular response to hypoxia; regulation of apoptotic process	[29, 42, 63]
P40261	NNMT	Nicotinamide *N*-methyltransferase	T,O	NAD biosynthesis via nicotinamide riboside salvage pathway	[20, 37, 40, 49, 103, 118]
Q01813	PFKP	ATP-dependent 6-phosphofructokinase, platelet type	T	Canonical glycolysis	[20, 37, 42]
P07737	PFN1	Profilin-1	T	Positive regulation of epithelial cell migration	[20, 46, 49]
P14618	PKM	Pyruvate kinase PKM	T,P	Canonical glycolysis; positive regulation of sprouting angiogenesis	[20, 26, 42, 81]
Q99541	PLIN2	Perilipin-2 (Adipose differentiation-related protein)	T	Regulation of lipid metabolic process	[37, 40, 42, 118]
P31949	S100A11	Protein A100-A11	T,U	Negative regulation of DNA replication; neutrophil degranulation	[20, 56, 93]
P06702	S100A9	Protein S100-A9	T,S	Innate immune response; neutrophil degranulation	[20, 42, 44, 80]
P63313	TMSB10	Thymosin beta-10	T	Actin filament organization; regulation of cell migration	[20, 56, 61]
P19320	VCAM1	Vascular cell adhesion protein 1	T,P	Cell adhesion; regulation of immune response	[20, 78, 81]
P08670	VIM	Vimentin	T	Cellular response to interferon-gamma; SMAD protein signal transduction	[20, 29, 40, 42, 62, 63]

*T* tissue, *P* plasma, *S* serum, *U* urine, O other

^a^ Subset of Uniprot gene ontology (GO) assigned biological processes (https://www.uniprot.org/)

### ccRCC tissue profiling

ccRCC accounts for the majority of RCC cases and as a result studies focused on profiling ccRCC are far more numerous than studies examining other RCC histologies. Potentially confounding the summarization of proteomic experiments in this review is the classification of ccRCC as RCC in several studies, resulting from RCC initially being divided into two major histologies prior to 2004 and ccRCC being viewed as conventional RCC [24, 25]. For the purposes of this review, we will consider studies describing the characterization of kidney tumors as RCC to be ccRCC unless additional information related to the genomic background, pathological classification, or anatomical location would suggest a different histology.

Two-dimensional electrophoresis (2-DE) is an early proteomic technology, utilizing a dual separation approach based on a protein’s isoelectric point and molecular weight on a polyacrylamide gel. The individual spots visualized by this methodology are then excised and subjected to mass spectrometry analysis to identify and annotate the protein spots, with differences in protein spot intensity between experimental conditions inferring differential protein abundance and identification of dysregulated proteins. Multiple studies have leveraged this technique to identify differential expressed protein profiles between ccRCC and NAT samples, followed by immunoblotting or immunohistochemical validation in independent samples [26–30]. Perroud et al. [26], identified 31 proteins that were differentially expressed between ccRCC tumor tissues and NATs derived from four patients, validating the overexpression of HSP27 (HSPB1) and PKM2 via immunoblotting. Pathway analysis indicated most of the differential expressed proteins were annotated as metabolic-related, prompting their investigation of the metabolite profiles in pooled urine samples, which revealed increased abundance of glycolysis by-product sorbitol. Raimondo et al. [27] also showed metabolic profiles are altered in ccRCC, and found immune response to be up-regulated in tumor tissues. Validation focused on the proteins that were up-regulated, with the authors verifying overexpression of ANXA2, LGALS1, PPIA, and FABP7. ANXA2 overexpression was primarily associated with the plasma membrane, and prompted several follow-up studies by this same group focused on delineating differences in protein abundance of plasma membrane domains between tumors and NATs [31, 32]. In addition to identifying several other proteins of interest, including VDAC1, BSG, and THY1, these studies described a strategy of annotating the cell surface proteome of renal cancer cells that may have applications in identifying novel therapeutic targets [33]. In another study, comparison of nine cases of ccRCC (paired tumor tissue and NAT) revealed the differential expression of 34 proteins, including the protein NDRG1, which was found to be elevated in ccRCC tissues, with subsequent validation experiments via IHC revealing the protein’s nuclear localization (versus membrane localization) was associated with a favorable prognosis, and functional assays revealing a potential tumor suppressor role of NDRG1 in ccRCC [29].

There are several inherent disadvantages of 2-DE profiling, including issues related to reproducibility, solubilization and visualization of hydrophobic proteins, low-throughput, and a narrow dynamic range of protein expression detection [34]. To circumvent these drawbacks, Multidimensional Protein Identification Technology (MudPIT) was developed, which relies on proteolytic digestion of proteins into peptide products and subsequent fractionation prior to mass spectrometry analysis. This “shotgun proteomic” approach facilitates several strategies for quantitation, including label-free quantitation (LFQ), and isobaric reporter tag labelling of peptides. Reports leveraging LFQ to profile ccRCC have mirrored earlier 2-DE studies in terms of the altered cellular pathways resulting from ccRCC pathobiology, albeit identifying far more differentially expressed proteins between tumors and NATs [35, 36]. Atrih et al. [37] used LFQ to examine the disparate protein profiles between ccRCC tissues and NATs, revealing almost 600 proteins to be differentially expressed. Using IHC, the authors validated the expression pattern of two proteins, CORO1A and ADFP, which were shown to have increased in abundance in ccRCC samples. Interestingly, the authors found that CORO1A was not overexpressed in renal cancer cells, but instead in the infiltrating lymphocytes localized in the tumor microenvironment, whereas ADFP overexpression was associated with tumor cells. This latter result highlights one drawback of bulk tissues analyses that many proteomic studies employ, specifically the loss of spatial information related to protein expression, as well as the mixing of different cell populations during sample homogenization.

Isobaric labelling, including technologies such as isobaric tags for relative and absolute quantitation (iTRAQ) and tandem-mass-tag (TMT), offer a strategy to reduce the time needed for data acquisition via sample multi-plexing [38]. Following proteolytic digestion, samples are labelled with a mass tag reporter ion that when subjected to fragmentation in the mass spectrometer, enables the measurement of peptide (and subsequently, protein) abundance across multiple samples [39]. An early report applied this approach, identifying a total 324 differentially expressed proteins between a tumor and NAT sample, with 99 robustly detected in replicate analyses [40]. Included in their iTRAQ experiment were two “control” samples, a transitional cell carcinoma (TCC) case and a kidney tissue sample from a patient with end stage glomerulonephritis, facilitating a comparison of other renal disorders and ccRCC. Although the authors examined their dataset in relation to several previous publications and validated several proteins, including VIM, SERPING1, NNMT, and LDHA via IHC or immunoblotting, they did not report the expression pattern of these proteins in their “control” samples, thus missing the opportunity to identify ccRCC-specific protein candidates. In another study that incorporated sample fractionation for deeper profiling, a larger cohort of patients were examined using the iTRAQ approach, wherein a pooled reference sample was included to link multiple iTRAQ sample plexes. In this proteomic-based discovery phase, the authors identified 55 proteins differentially expressed between ccRCC and NAT samples [41]. From this list of proteins, the authors prioritized candidates that had gene ontology (GO) annotation as “secretory” and could serve as potential serum biomarkers, validating five proteins—ENO1, HSPE1, HSPB1, AHNAK, and LDHA via immunoblotting and/or IHC tissue microarray (TMA) profiling. Mirroring their proteomic results, the proteins ENO1, HSPB1, LDHA, and AHNAK were elevated in ccRCC, while HSPE1 was down-regulated. Of note, the authors did not verify any of these candidates in serum samples, missing the opportunity to link tissue protein profiles to circulating serum protein profiles.

Moving beyond comparisons of ccRCC tumor and NATs tissues only, several studies have sought to delineate protein expression patterns associated with disease severity, including stratifying patient samples based on tumor grade, tumor stage or comparisons of primary and metastatic lesions. Perroud et al. [42] leveraged a LFQ approach to characterize the proteome of fifty kidney tissues comprising equal number of normal tissues and Fuhrman grades 1–4 of ccRCC tissues. Prior to their evaluation of archival formalin-fixed paraffin embedded (FFPE) tissues, the authors first assessed whether the FFPE process introduced any bias in the proteomic data, finding only a few proteins displayed varied abundance between sample matched frozen tissues. Following this preliminary result, the authors then examined the 50 FFPE tissues, wherein they identified 105 proteins that showed significant differential abundance across the four grades and normal tissues. The protein NPM1, which has been shown to impact nucleolar morphology, showed increasing expression from normal tissue and G1 tumors to G2, G3, and G4 tumors. Considering the Fuhrman grading system is based on the nucleolus morphology and sizing, the increasing expression of NPM1 in higher grade tissues served as a robust, positive control. Pathway analysis of the differentially expressed proteins revealed higher expression proteins involved in glycolysis (PGK1, ALDOA-C, ENO1) xenobiotic metabolism (ALDH4A1, ALDH1A1, ALDH9A1), and down-regulation of proteins associated with apoptosis (AIFM1) were associated with higher tumor grade and are representative of the cellular mechanisms involved in ccRCC progression. Interestingly, the authors found that proteins clusters could differentiate G1/2 tumors and G3/4 tumors, mirroring the disparate prognosis of patients and morphological differences observed between low grade ccRCC versus high grade ccRCC [43]. Another study examining proteome profiles associated with tumor stage in ccRCC using a modified 2-DE approach (2D-DIGE), with the resulting protein profiles visualized using a principle component analysis (PCA) showing a clear separation between non-neoplastic kidney tissues and pT1, pT2, and pT3 staged tumors [44]. Similar to increasing tumor grade, increasing ccRCC tumor stage associated with alterations in metabolism, specifically up-regulation of glycolysis with a parallel decrease in TCA cycle protein expression. The authors highlighted and validated the differential expression of several select proteins, PHB, PRDX3, and S100A9, using IHC and immunoblotting. While PRDX3 down-regulation was associated with increasing tumor stage, S100A9 and PHB increased in abundance and were also associated with tumor grade. An interesting feature was detected in the immunoblot results for PHB, wherein the expected 30 kD band showed minimal difference across the four experimental conditions, while a slightly larger band at 40 kD was discriminatory and may suggest a post-translation modification on PHB increased in higher stage disease. To investigate the protein alterations associated with metastatic disease, Laird et al. [45] used an immunofluorescence antibody-based approach to profile a TMA composed of 138 ccRCC tissues, 14 papillary tissues, 103 renal vein tumor thrombus (VTT) tissues, and 69 metastatic tissues. Evaluating a panel of antibodies comprised of MKI67, TP53, VEGFR1 (FLT1), VEGFD, SNAIL (SNAI1), and SLUG (SNAI2) paired with a technology called Automated Quantitative Analysis (AQUA) that allows for an unbiased assessment of protein expression, the authors showed all the proteins, except for VEGFD, were significantly elevated in metastatic disease relative to VTT and primary lesions. Although VEGFD did not show an association with disease severity, both VEGFD and VEGFR1 expression were prognostic in primary tumors, with elevated expression associated with reduced cancer specific survival. Less clear was any discriminatory value for these markers between ccRCC and papillary primary tumors, which would be relevant in the context of ccRCC displaying elevated angiogenic signaling resulting from a pseudo-hypoxia phenotype due to VHL inactivation. Using an iTRAQ-based approach, Masui et al. [46] characterized six cases of ccRCC tissues and patient-matched NATs, and six unmatched metastatic tissues, identifying 29 proteins that were differentially expressed between primary and metastatic lesions. Three proteins, LGALS1, PFN1, and YWHAZ, were selected for validation via immunoblot in the same patient cohort and IHC using TMAs derived from an independent cohort, which showed increased expression in primary ccRCC relative to NATs, and increased expression in metastatic lesions relative to primary tumors. An additional assessment was done to determine the prognostic value of these three candidate proteins which showed PFN1 was associated with a poor prognosis. In a follow-up study, the same team performed a deeper investigation of this proteomic dataset to identify the cellular pathways associated with metastasis, revealing the metabolic pathways glycolysis, pyruvate metabolism, and the TCA cycle to be highly dysregulated [47]. They validated the proteomic results using a PCR array of metabolic genes and found a high degree of concordance for select pathways between the two datasets. Overall, these studies reveal that many of the cellular processes that are dysregulated in early stage ccRCC tumors, in particular metabolism-related pathways, are maintained and amplified during disease progression [48].

With the advent of high-throughput technologies for genomic, transcriptomic, and proteomic characterization, several studies have begun to incorporate multiple levels of molecular information to facilitate “multi-omic” profiling of tissue samples. Integration of multiple omic data types allows for researchers to begin to link genomic alterations to observed phenotypes, as well as identify aberrant regulatory mechanisms that may not be detected with a single omic dataset. Leveraging a proteotranscriptomic approach, Neely et al. [49] performed a series of comparative transcriptomic and proteomic experiments to identify altered gene profiles associated with the molecular phenotype of ccRCC. The authors identified 342 proteins that were differentially expressed between ccRCC and NATs samples, with subsequent pathway analysis results further supporting ccRCC to be defined as disease with significant metabolic dysfunction. A subset of the samples were previously characterized at the transcript level, and integration of both data types revealed ~ 70% of the transcripts and proteins showed a positive correlation, ~ 94% of the differentially expressed mRNA–protein pairs showed a positive correlation, although the authors noted this correlation was not linear. In this same report, the authors also identified several candidate proteins associated with aggressive ccRCC, including CFL1, PFN1, NNMT, and ALDOA that were found to be elevated in Stage 4 disease. Interestingly, in this particular dataset, CFL1, PFN1, and ALDOA were found to be decreased in early stages relative to NATs. In another study, FFPE ccRCC tissues and patient matched NATs that were previously characterized at the transcript level, were characterized at the protein level, wherein the authors found disparate cellular pathways over-represented at each biological domain [50]. At the protein level, metabolic pathways were robustly shown to be altered, whereas transcriptomics indicated immune response pathways were more significantly impacted. The antigen presentation pathway was observed to be dysregulated in both datasets, with CD74 showing significant differential expression at both the mRNA and protein levels. Specific to the proteomics results was the observed down-regulation of SIRT3, which was not detected at the transcriptomic level, and highlights the added benefit of employing complementary technologies for molecular characterization. Recently, our lab led the Clinical Proteomics Tumor Analysis Consortium (CPTAC) effort to interrogate ccRCC tumors using a proteogenomic approach, comprehensively profiling and integrating genomic, epigenomic, transcriptomic, proteomic, and phosphoproteomic measurements [20]. In addition to delineating novel features of ccRCC at the genomic level, including the identification of a subset of tumors displaying a high degree of chromosome instability and chromosomal translocation involving 3p as a prominent event, we also showed there are four major subtypes of ccRCC defined by disparate tumor microenvironment compositions and proteomic signatures. These four subtypes—CD8+ Inflamed, CD8− Inflamed, VEGF Immune Desert, and Metabolic Immune Desert—were not only predicted to have distinct responses to immune checkpoint and anti-VEGF therapies, but also predicted patient survival. In an effort to expand therapeutic options available to ccRCC patients in the clinical setting, we leveraged our phosphoproteomic results to prioritize the identification of phospho-substrates of kinases with current FDA-approved inhibitors, revealing signaling associated with MAPK/ERK, PI3K/AKT/mTOR, and G2/M cell cycle stalling to be elevated in ccRCC tumors and potential druggable targets. Global proteomic results revealed the up-regulation of immune response pathways and hypoxic signaling, as well as the previously described alterations in cellular metabolic pathways. Interestingly, we showed that the down-regulation of oxidative phosphorylation was only detectable at the protein level, and not the mRNA level, which not only highlights the added benefit of multi-omic profiling to gain unique insight into ccRCC biology, but also cautions against using mRNA levels as a surrogate for protein expression. The continual observation of altered metabolic profiles in ccRCC prompted Zhang et al. [51] to examine alterations lysine succinylation between ccRCC and NATs, which has been previously linked to metabolism regulation and is an abundant PTM on mitochondrial proteins [52]. Employing a TMT quantitation strategy and antibody-based enrichment of succinylated peptides, the authors integrated global and succinlyome measurements, finding many of the abundances changes of succinylated peptides were the result of global protein changes. Together, these results show the utility of multi-omic profiling using complementary technologies to deepen our understanding of ccRCC, and the unique insight that can be gained when integrating measurements at various levels of gene expression and regulation.

IHC serves as an orthogonal methodology to validate protein expression patterns and enables researchers to determine the spatial distribution of a protein across different areas of the tumor and surrounding tumor microenvironment. Although IHC multiplexing technologies are emerging [53], the number of proteins that can be simultaneously profiled is still relatively low. MALDI Mass Spectrometry Imaging (MALDI-MSI) is an established technique that greatly expands the types of biomolecules that can be directly measured, including proteins, peptides, lipids, glycans, and small molecules, and links these molecular profiles to specific anatomical locations [54, 55]. Oppenheimer et al. [56] used MSI to assess the tumor margin of ccRCC resected tissues from 75 patients and determined the distribution of protein abundance across four distinct regions: tumor, tumor-margin, NAT margin, and NAT. Their results robustly showed the up-regulation of S100A10, S100A11, MIF, and TMSB10 in tumors, while mitochondrial proteins such as COX6C, COX5B, COX7C, UQCRG, and CYCS were down-regulated, mirroring previous studies examining lysed bulk tissue samples. Interestingly, the authors found many of the down-regulated proteins were also dysregulated in the tumor margins of the resected tissue, suggesting that molecular alterations precede changes in cell morphology, a feature that has been well established in other tumor types [57–60]. Utilizing MALDI-MSI, another study examined the spatial distribution of protein and lipid profiles across ccRCC tumor and NAT samples to delineate differential abundance of these biomolecules [61]. Proteins significantly up-regulated in tumors included TMSB10, TMSB4X, HBB, HBD, HBG1, and FABP7, while COX5B, COX5A, FABP1 were down-regulated. This particular cohort of patient samples included information related to disease recurrence, facilitating the identification of protein profiles that could distinguish non-recurrent and recurrent ccRCC. Only two proteins—DEFA1 (elevated in tumors) and LYZ (significantly decreased in tumors)—could robustly differentiate disease recurrence, whereas the authors found lipid profiles were more discriminatory for disease recurrence in addition to discriminating tumor versus NAT. To determine if MALDI-MSI could delineate protein expression patterns associated with tumor grade, Stella et al. [62] examined 14 samples from 13 patients with various intra-tumor histological grades. The resulting spectra showed a clear separation of tumor and NAT regions via PCA, in addition to distinguishing normal cortex tissue from normal medulla tissue. Only G1 tumors could be robustly separated from G4 tumors. Further investigation of the features that could discriminate G1 and G4 tumors found the identified peaks were derived from VIM, which was elevated in G4 tumors and histone subunits H2A and H4, which were elevated in G1 tumors. These studies show the complementary results of MSI approaches to current proteomic characterization techniques, with the added benefit of gaining information related to the spatial distribution and localization of molecules of interest across resected tissues samples.

### Multiple RCC histology tissue profiling

With substantial evidence of the disparate genomic backgrounds of the various histological subtypes of RCC [11], identifying discriminatory protein features would be relevant in confirming pathological annotation, as well as understanding the contrasting prognosis associated with each RCC subtype. Towards this goal, an early report from Lichtenfels et al. [63] first profiled ccRCC and paired NAT samples to identify differentially expressed proteins using a 2-DE approach. A total of 248 proteins were found to be differentially expressed, with three proteins—CALB1, GSN, and FABP3—selected for examination in a TMA panel comprised of ccRCC (n = 40), pRCC (n = 31), chRCC (n = 16), and renal oncocytic lesions (n = 9) with corresponding NATs. CALB1 staining was weak in many of the RCC tissue samples, but robustly detected in oncocytic and NAT regions indicating negative CALB1 expression was a robust marker of malignant renal disease. GSN expression in NAT was primarily detected in distal tubule and collecting duct cells, while proximal tubule and glomeruli cells were negative. In RCC tissues, almost a third of ccRCC and pRCC tissues were negative for GSN, while > 50% of chRCC and oncocytic tissues were positive for GSN. This pattern of nephron cell type specificity for GSN expression seemed to reflect the cells of origin from which chRCC and oncocytomas arise, specifically distal tubule and collecting duct cells [13]. FABP3 showed the most promise for discriminating the various RCC histological subtypes, with FABP3 expression highest in chRCC and oncocytic RCC lesions, negative in pRCC tissues, and heterogeneous in ccRCC, with approximately 40% of samples showing positive staining. Valera et al. [64] used a very similar experimental design, wherein they utilized 2-DE based approach to identify the differential protein patterns associated with ccRCC, pRCC, chRCC, and renal oncocytomas relative to corresponding NATs. The authors highlighted proteins that showed significant differential expression between NATs and ccRCC (HSPB1, TPI1, HBB, APOA1, and PRDX2), chRCC (SOD1, RAD23B, and SERPINA1), and oncocytic lesions (ENOA1), whereas no differentially expressed proteins were identified between pRCC and patient matched NATs. HSPB1 and TPI1 were selected for IHC validation in ccRCC tissues, with the staining pattern concordant with the proteomic analysis results; however, the authors missed the opportunity to further assess the expression of these proteins in other RCC lesions. Considering several of the proteins (ENOA1 and SOD1) have been associated with ccRCC in previous reports [35, 47], the utility of several of the candidate markers may be negligible for discriminating various RCC histologies. Using an antibody panel composed of MKI67, CRP, CA9, HIF1A and HIF2A, Abel et al. [65] sought to assess the expression patterns of these proteins in a TMA comprising ccRCC (n = 42), pRCC (n = 11), and chRCC (n = 1) tumors and NATs, and link these results to clinicopathological outcomes. Although CA9, HIF1A and HIF2A were elevated in tumors, the observed differential expression was not determined to be significant. CRP was shown to be reduced in tumors, whereas MKI67 was elevated and found to associate with disease recurrence. Similar to the previous report, there was no assessment of differential abundance of this antibody panel across the three RCC histologies; albeit with only one case of chRCC, interpretation of any potential results would be minimal. Oncocytomas and chRCC are both thought to arise from the distal tubule epithelium [66], and Drendel et al. [67] profiled oncocytoma and chRCC FFPE samples to identify markers that would discriminate between benign and malignant RCC lesions. PCA showed the resulting protein profiles were clearly separated, with 51 proteins and 59 proteins enriched in oncocytoma and chRCC tissues, respectively. Two proteins—LAMP1 and ITGAV—were selected for verification in an independent cohort of oncocytomas and chRCC, with ITGAV1 expression to exhibit strong staining in oncocytomas, whereas LAMP1 staining was robustly detected in chRCC.

Two studies explored the phosphorylation patterns in RCC using antibodies targeting phosphorylated substrates in RCC cell lines, and primary and metastatic tissues [68, 69]. Lin et al. [68] constructed a TMA comprised of 70 ccRCC, 40 pRCC, and 18 chRCC primary tumors, 22 metastatic RCCs, and 24 NAT and probed phospho-substrates associated with mTOR signaling (AKT-S473, mTOR-S2448, and p70S6-T389). Almost all of the malignant samples showed strong positive staining for these various phospho-substrates relative to NATs indicative of constitutive mTOR signaling and providing robust evidence for the rationale of selecting mTOR inhibitors for treatment of RCC. Of note, the authors did acknowledge the mixed efficacy of targeting mTOR in the clinical setting and need to identify other cellular pathways that may contribute to mTOR inhibition resistance. Haake et al. [69] leveraged a pan-tyrosine enrichment approach to identify activate signaling cascades in ccRCC and pRCC tumors and RCC cell lines. Their results showed PTK2 phosphorylation to be ubiquitous across all RCC samples analyzed, with a tyrosine kinase inhibitor screen consisting of 63 compounds showing those targeting PTK2 to have the most robust response in vitro. Interestingly, when evaluating differential expression of phospho-substrates in ccRCC and pRCC tumors, EGFR-Y1197, ERK2-Y187, ERK1-Y204, and TENC1-Y483 were elevated in ccRCC, while DDR1-Y792/6 and PP4B-Y849 were elevated in pRCC. These latter results suggest disparate signaling pathways activated in ccRCC and pRCC, respectively, and provide rationale for exploring targeting these signaling cascades as a therapeutic intervention in these RCC subtypes.

## Biological fluid profiling

Tissue biopsy sampling is a routine procedure that enables clinicians to obtain a small portion of the malignant tissue to investigate the histopathological and molecular features for diagnostic and prognostic information. However, tissue biopsies are considered an invasive procedure and due to the limited sampling area, may not be fully representative of the tumor, which are known to be heterogeneous [70]. Utilizing biological fluids as a liquid biopsy would offer a minimally invasive strategy for repeat sampling to monitor disease progression and possibly be more representative of the molecular features associated with tumorigenesis [71]. With the ultimate goal of identifying candidates for disease diagnosis and early detection in RCC (Table 1), many studies have applied proteomic approaches to characterize serum/plasma and urine protein profiles.

### Serum/plasma profiling

Of the multiple biological fluids found in the human body, blood is often considered to be the ideal source for protein candidates. The relatively non-invasive, simple procedure involved in specimen collection would circumvent the challenges and expertise involved in tissue biopsy acquisition, and the network of arteries, veins, and capillaries that come in contact with organs offers a means for proteins that are secreted, shed, or otherwise released by tumor tissues to be circulated [72]. Several inherent challenges in characterizing protein profiles in serum or plasma, include the dynamic range of protein concentration, which spans up to 12 orders of magnitude, as well as the presence of a small number of highly abundant proteins that mask more low abundant proteins [73, 74]. To address this, a variety of strategies have been performed to investigate and identify differentially abundant proteins related to RCC in plasma or serum samples, including immunodepletion to remove highly abundant plasma proteins (i.e. albumin, transferrin, haptoglobin) and protein pre-fractionation [75]. One study used 2-Dimensional Image Converted Analysis of Liquid chromatography mass spectrometry (2DICAL) to profile plasma samples from twenty RCC patients and 20 healthy controls [76]. The 2DICAL strategy is equivocal to shotgun proteomic approaches, and the resulting profiles showed that FN1 was elevated in RCC patient plasma relative to controls. Considering many proteins that are secreted or localized to the plasma membrane are N-linked glycosylated, thus having a higher chance of being shed into the extracellular space, multiple studies have included N-linked glycoprotein enrichment strategies for biomarker detection [77]. Gbormittah et al. [78] characterized global proteome, N-linked glycoproteome, and N-glycome plasma profiles of RCC patients before and after nephrectomy using a combined approach of immunodepletion and lectin enrichment. The author’s global proteomic results revealed several proteins (i.e. HSPG2, CD146, VCAM1) associated with metabolic processes, immune response, and various signal transduction pathways were all reduced following surgical intervention. Glycoproteomics identified another subset of proteins that were reduced after nephrectomy, such as APOB, LGALS3BP, and FN1, while glycomics indicated sialylation and high mannose glycan structures were associated with pre-treatment RCC plasma profiles. In another study, the authors focused on delineating alterations in the serum proteome associated with early stage ccRCC using a paired immunodepletion and iTRAQ approach [79]. Pooling serum samples from ten ccRCC patients and ten healthy controls, they identified 30 differentially abundant proteins, with HSC71 (HSPA8) showing greatest abundance difference. The authors then validated HSC71 expression using ELISA, profiling the serum of ccRCC patients, healthy controls, and patients with other urological diseases such as angiomyolipoma of the kidney, benign prostatic hyperplasia, urinary tract infection, and urolithiasis, showing HSC71 was elevated in ccRCC patient serums relative to healthy controls and non-ccRCC patients. An independent study examined the serum of a larger cohort of patients that included twenty-nine early stage ccRCC patients, 20 patients with transitional cell carcinoma, 24 patients with benign kidney neoplasia, 18 healthy controls, and eight patients with prostate cancer [80]. A total of 74 proteins were found to have differential abundance between ccRCC and healthy controls serum samples, and 27 proteins that were differentially abundant between ccRCC and the other three groups. Leveraging the results from the TCGA characterization of ccRCC, the authors sought to link their serum profiles to tissue gene expression profiles, identifying 11 proteins, including C1QB, C1QC, ANXA1, LYZ, S100A9, and SERPINA4, that were differentially abundant in both datasets and were associated with RCC tumor stage and grade. An important caveat of many of these studies is that few have directly linked the resulting plasma/serum profiles to RCC tissue signatures. An exception was an early report that described a combined tumor-plasma proteome analysis on a single patient, examining tumor tissue, NAT, and pre-operative plasma sample [81]. To identify candidates of interest, the authors prioritized proteins that met four criteria: (1) identified in tumor tissue, (2) not identified in NAT, (3) identified in plasma, and (4) higher abundance in tumor relative to plasma. This approach and filtering criteria identified eight proteins of interest—CDH11, PKM (KPYM), VCAM1, CDH5, DDX23, WWC1, CHD4, and NCOA6—with subsequent validation of the presence of CDH5, CDH11, DDX23, and PKM via immunoblotting in the plasma of the same patient and four others. Although samples analyzed were from only one patient in the initial proteomic characterization, the overall experimental design is ideal for the identification of potential candidate protein biomarkers. This approach would not only determine the differential expression of proteins between tumor and NAT, but also identify tumor-related proteins that are detectable in the circulation.

Several reports have focused on examining “biological trash” in circulating profiles, specifically focusing on examining low molecular weight endogenous peptide fragments. Peptidome profiling is thought to offer a higher degree of sensitivity and specificity relative to other approaches, relying on the aberrant biological activity of disease-related proteases, enzymatic reactions, and degradation products [82]. Using C18-functionalized beads to enrich peptide fragments paired with MADLI-TOF data acquisition, Gianazza et al. [83] profiled the serum of eight-five ccRCC patients, 92 controls, and 29 patients with histologically defined non-ccRCC. Clustering patients into three groups: malignant tumors, benign renal masses, and healthy controls, resulted in the identification of 5 peptides that were discriminatory for the three groups. Incorporating the results of subsequent ESI-LC–MS–MS analysis allowed for the identification of the endogenous peptides, revealing peptides from SDPR and ZYX to be decreased and peptides from SRGN and TMSL3 to be increased in the serum of ccRCC patients. Huang et al. [84] also performed peptidome profiling of serum samples derived from RCC patients and healthy controls, identifying 19 peptides that were differentially abundant between the two groups. Of these, four peptides showed high specificity for discriminating RCC and controls, with three trending downward in abundance, and one trending upward. Highlighting one drawback of peptidome profiling, only two of these four peptides could be identified, derived from the proteins CUBN (decreased) and APOA1 (increased), respectively. In an attempt to identify peptidome profiles associated with RCC, Kodera et al. [85] examined plasma samples from RCC patients before and after nephrectomy. Although the authors were able to find a peptide reduced in abundance following surgical intervention and show it was specific to RCC relative to bladder cancer, the authors were unable to annotate the protein it was derived from. In a larger cohort that examined serum samples from 64 healthy controls and 78 ccRCC patient, including 20 that had pre- and post-nephrectomy serum samples, Yang et al. [86] identified 24 peptides that were differentially abundant across all groups. Three peptides were found to up-regulated in ccRCC that then returned to levels similar to healthy controls following nephrectomy, derived from the proteins RBP6, TUBB, and ZFP3. Together, these studies suggest peptidome profiling has the potential to elucidate candidates of interest; however, the minimal overlap between independent studies and lack of validation in independent cohorts suggest this methodology warrants further development.

### Urine profiling

The kidneys function to balance electrolyte levels, regulate blood pressure, as well as filter circulating blood to remove waste, resulting in urine profiles mirroring the physiological status of an individual. Relative to plasma, urine has a narrower dynamic range of protein concentration, and the reduced abundance of proteins, such as albumin, transferrin, and haptoglobin, enables the detection of lower abundant proteins [87, 88]. With respect to malignancies associated with the kidney, prostate, and bladder, urine is a proximal biological fluid that may offer a richer source of proteins of interest relative to blood. Albeit, the composition of urine, which includes proteins, urea, inorganic salts, and other biomolecules, presents a unique challenge for sample processing prior to proteomic characterization; however, a myriad of techniques have been developed to isolate or enrich proteins from urine, including analytical ultracentrifugation, precipitation, ultrafiltration, and tip-based approaches [89–91]. Sandim et al. [92] examined the urine of ccRCC patients, grouped by prognosis (good versus poor) and healthy controls. Pooling the urine samples of each group and then performing ultrafiltration as a proteomic-compatible preparatory step, the authors applied a multi-faceted approach that included 1-DE, 2-DE, and LFQ to identify differentially abundance proteins across the three sample classifications. Qualitative assessment via 1-DE revealed the proteins CO3, FIBG, MGAT4A, and APO1A to only be detected in ccRCC samples, while CDH13, AMYA, and APOD were only identified in the urine of healthy controls. Quantitative assessment via LFQ or 2-DE profiling revealed the increasing expression of APOA, FN, HP, and MGAT4A in controls, good prognosis ccRCC samples, and poor prognosis samples, with a concordant decrease in abundance of the proteins KNG1, UMOD, APOD, UBC, CD59, and HSPG2. Chinello et al. [93] stratified ccRCC patients based on the degree venous infiltration of RCC tumors, as assessed by Computed Assisted Tomography, generating three distinct groupings: vascular infiltration, renal vein infiltration, and renal vein thrombosis. Three proteins—UMOD, RALA, and CNDP1—displayed decreased expression proportional to RCC infiltration, while 26 proteins (i.e. HP, LUM, CRNN, ANXA2) were increased in abundance in the pooled patient samples. The authors also examined plasma protein profiles from these same patients, and unlike the urine proteome alterations, no proteins showed concordance with the degree of increasing vein infiltration, while two were inversely correlated—APOA1 and K2C1. When interpreting this latter result, the authors acknowledged a high degree of overlap between the datasets derived from different biological fluids in terms of differentially abundant proteins, but the loss of discriminatory proteins for disease severity in plasma samples may be related to the loss of kidney function or architecture during renal oncogenesis. Santorelli et al. [94] also sought to identify urinary proteins that could differentiate disease severity, focusing on alterations in the N-linked glycoproteome profiles. Employing the N-glyco-FASP technique [95], which uses lectin enrichment of glycopeptides prior to mass spectrometry analysis, the authors examined ccRCC patients with early stage disease (pT1) and late stage disease (pT3), as well as healthy controls. Generating three patient urine pools and precipitating the protein component, the resulting profiles showed a trend of protein expression associated with ccRCC stage. Three proteins—CD97, COCH, and P3IP (PIK3IP1)—showed elevated abundance in the urine of low-stage patients relative to healthy controls, and then increasing abundance in urine from low stage to high stage. Proteins found to be decreased in abundance in low stage ccRCC relative to healthy controls included APOB, FINC, CERU, CFAH, HPT, and PLPT; however, these proteins levels were slightly elevated in the urine of pT3 ccRCC patients, albeit still lower than levels in healthy controls. In the latter study, the authors highlighted the benefit of glycoprotein enrichment, which facilitated the detection of several proteins that might have otherwise not been identified and quantified. Other studies have sought to differentiate the urine protein profiles of ccRCC patients from those of other renal disorders, including patients with oncocytomas or hereditary VHL mutations. Mandili et al. [96] used a 2-DE approach to identify proteins that could discriminate patients with sporadic ccRCC from those with VHL disease (VHLD) and healthy controls, respectively. The authors performed several paired analyses to elucidate the differentially expressed proteins between healthy controls, patients with sporadic ccRCC, and VHLD patients, including subsets of those VHLD patients with and without a history of ccRCC. While the authors identified proteins that differentiated sporadic ccRCC and VHLD patients from healthy controls, as well as sporadic ccRCC patients from VHLD patients with distinct ccRCC diagnoses backgrounds, the authors chose to validate two proteins—A1AT and APOH—which were found to be elevated in the urine of VHLD-ccRCC-positive patients relative to the other three groups. Considering both ccRCC and VHLD share a similar genotype profile (loss of *VHL*), delineating differences in urine proteome profiles would be challenging. However, the unique expression pattern of the proteins highlighted by the authors (A1AT and APOH) might suggest a disparate progression of diseases between VHLD patients with ccRCC and those with sporadic ccRCC. Another report focused on identifying proteins that would differentiate healthy controls, patients with ccRCC, and patients with oncocytomas [97]. In a discovery phase, the authors pooled urine samples from healthy controls, oncocytomas, and ccRCC patients with progressive disease and non-progressive/early stage disease (pT1a, tumor size ≤ 4 cm). A total of 131 proteins showed differential abundance between healthy controls and ccRCC patients with early stage disease, while 71 proteins were differentially abundant between ccRCC patients with early stage disease and oncocytomas. The authors sought to verify the abundance profiles of several proteins in an independent cohort using parallel reaction monitoring (PRM), finding concordance of increased abundance of GLRX, CST3, SLC9A3R1, HSPE1, FKBP1A, and EEF1G in early stage patients relative to healthy controls, and increased abundance of C12orf49 and EHD4 in early stage disease urine samples relative to oncocytomas. When comparing early stage urine samples to those from patients with higher staged disease (progressive ccRCC group), five proteins were elevated EPS8L2, CHMP2A, PDCDPI6, CNDP2, and CEACAM1, with authors finding the combined urinary abundance of EPS8L2 and another protein, CCT6A, to have prognostic value in ccRCC.

In addition to investigation of urinary proteome in ccRCC, peptidome profiling has also been examined. With evidence of disparate endogenous peptide profiles between plasma and urine [98], potentially resulting from the physiological activity of the kidney, exploration of the peptidome in urine is warranted. Frantzi et al. [99] using capillary electrophoresis coupled with mass spectrometry analysis (CE-MS) profiled forty RCC cases and 68 non-diseased controls; the latter group included normal controls, non-RCC reference patients, and patients with pre-disposing factors towards RCC. In this training phase, the authors revealed 86 peptides (40 which were subsequently identified) with differential abundance between the two groups. In a validation cohort that comprised of RCC cases (n = 30), non-diseased controls (n = 46), and additional patient groups consisting of diabetic nephropathy (n = 195), focal segmental glomerulosclerosis (n = 54), membrane glomerulonephritis (n = 65), systemic lupus erythematosus (n = 46), IgA nephropathy (n = 126), vasculitis (n = 121), cardiovascular disease (n = 33), and bladder cancer (n = 219), the authors found their 86 peptide classifier was discriminatory for RCC against all groups except for vasculitis (64% specificity) and bladder cancer (76% specificity). Interestingly, when exploring the identified peptides, the authors found the resulting profiles to be reflective of the physiological dysfunction of the RCC microenvironment, including reduced abundance of > 1.4 kDa collagen fragments due to extracellular matrix remodeling in renal carcinogenesis and increased abundance of peptides from plasma proteins due to renal function dysregulation. Chinello et al. [100] also examined urinary peptidome profiles derived from healthy controls, ccRCC patients, histologically defined non-ccRCC patients, and renal benign masses. In the discovery phase, the authors found 12 urinary peptides that could discriminate patients with malignant disease from those with benign disease. In an independent cohort, the 12 peptide panel displayed 87% specific and 76% sensitivity for discriminating malignant and benign/control patients, with identification showing peptide fragments from the proteins SCTM1, UROM, MEP1A, KPB1, OSTP, and FIBA to be increased in abundance in malignant urine samples. In a follow-up study, the same group sought to link peptidome profiles to clinicopathological features such as grade, stage, and tumor size [101]. Examining urine samples from ccRCC patients and healthy controls, the authors identified 15 peptides that associated with tumor size and 9 that were differentially abundant relative to controls. A total of 26 peptides associated with tumor stage, including 15 that discriminated ccRCC and controls, and 5 peptides that associated with tumor grade, 4 of which were differentially abundant. Subsequent identification of the peptides found that a peptide fragment from C1RL was elevated in the urine of ccRCC patients, but showed decreasing expression in the urine of patients with higher grade disease, while GAPDHS, which was also reduced in ccRCC urine samples, showed increasing abundance in higher grade disease. Several stage associated peptides included fragments from FIBA and NOTCH2, with the latter showing no differential abundance between ccRCC and control urine samples. Overall, urine is a rich source of candidates of interest for RCC, however, the lack of any FDA-approved urinary markers for RCC suggests more work is to be done to prioritize candidates that may offer diagnostic and discriminatory benefit in the clinical setting.

### Profiling other biological fluids and sources

In addition to blood and urine profiling, several other biological sources have been characterized using proteomics. Interstitial fluid not only serves as transport medium for secreted proteins, nutrients and waste materials between cells and capillaries, but tumor interstitial fluid (TIF) could serve as a rich source of candidate markers due to the proximity of this biological fluid to the tumor [102]. Teng et al. [103] profiled the TIF obtained from ten patients with ccRCC and matched NATs. With previous reports have indicated that TIF contains a significant portion of highly abundant plasma proteins, the authors employed immunodepletion prior to analysis, resulting in the identification of 539 proteins, including 138 with differential abundance between ccRCC and NAT TIF samples. GO annotation revealed many of the proteins increased in abundance in ccRCC TIF localized to the plasma membrane, and included proteins previously identified in more distal biological fluids such as urine or plasma/serum. This supports the hypothesis that the TIF proteome is primarily comprised of shed or secreted proteins that are eventually found in the circulation. Eight proteins (NNMT, ENO2, TSP1, CD14, LGALB1, TBG (SERPINA7), ANXA4, and FTH1) were selected for validation, with increased abundance verified via immunoblotting, selective reaction monitoring (SRM), or ELISA in TIF samples or patient-derived serum samples. SRM showed robust concordance of increased expression of all selected protein in TIF samples, and increased abundance of CD14, TBG, and TSP1 (THBS1) in ccRCC serum samples relative to a healthy control serum samples, while ELISA showed elevated abundance of ENO2 and TSP1 in ccRCC patient-derived serum. Using fluid from a renal cyst, Minamida et al. [104] leveraged a 2-DE approach to identify differentially abundant proteins profiled in cyst fluid derived ccRCC tumors compared to cyst fluid derived from NAT. The authors identified over 200 proteins across the samples, and selected only one protein, YWHAB, for verification via immunoblotting due to its previous lack of association with RCC. Increased abundance of YWHAB was robustly observed in ccRCC cyst fluid and the urine from RCC patients, whereas exploration of tumor tissue and serum showed equal abundance. Validation in an independent cohort of urine from RCC patients (n = 89) and healthy controls (n = 76) via ELISA, showed elevated expression in RCC urine samples and a corresponding decrease in the urine of RCC patients following nephrectomy. The authors did mention several caveats regarding the utility of the candidate protein, including the lack of detection of YWHAB in the urine of all RCC patients, detection of YWHAB in the urine of patients with other cancer types, and limitation of the marker as an indicator of malignancy, but not a marker to assess response to treatment.

Extracellular vesicles (EVs) are secreted microvesicles that have been shown to have a role in proximal and distal intercellular communication. EVs are comprised of several classes, including exosomes (30–100 nm) and ectosomes (100–1000 nm), with reports describing disparate mechanisms of cellular release into the extracellular space [105]. Several studies have shown that EVs are functionally active and can modulate recipient cell phenotypes via the transfer of proteins and nucleic acids (mRNA, miRNA, and DNA) [106–110]. Seeking to identify potential EV-based protein candidates for RCC, Raimondo et al. [111] performed a comparative proteomic analysis of exosomes derived from the urine of RCC patients and healthy controls. After verifying the presence of several EV-positive markers and morphological characteristics using electron microscopy, the resulting protein profiles were assessed using a qualitative approach, determining the disparate detection of select proteins in either RCC patient derived exosomes, or healthy control derived exosomes. Ten proteins were selected for validation, with proteins CD10, EMMPRIN, DPEP1, SDCB1, and AQP1 decreased in abundance in RCC-derived exosomes, and CP, MMP9, PODXL, CAIX, and DKK4 increased in abundance. Another report described the development of ex vivo models of ccRCC and NATs, and subsequent enrichment of EVs secreted from both tissue types [112]. In this manner, the authors could circumvent the challenge of delineating tumor-derived EVs from the total populations of EVs in a biological fluid, as well as examine a more clinically relevant model. Proteomics identified proteins that were only detected in one pathological EV condition, as well as 397 proteins that were differentially abundant. The protein, AZU1, showed the highest fold-change between RCC-derived EVs and NAT-derived EVs, and displayed increasing abundance in advanced ccRCC. Validation in the EVs derived from the sera of patients showed elevated levels in ccRCC relative to sera from healthy controls. Together, these studies are representative of the novel insight that can be gained by examining other sources that are not routinely collected in the clinical setting.

## Future directions

As we look to the future, there are still several aspects of RCC-related biology that have yet to be explored at the protein-level, as well as several unmet clinical needs. The continual utilization of established proteomics technologies, and the application of those currently in development will be the first step in addressing some of these areas that were not discussed previously in this review. Despite the multitude of studies examining the protein profiles of ccRCC (Table 2), studies focusing on rarer histological subtypes are less frequent, and large-scale, deep proteomic characterization of hundreds of RCC cases that include all the various histologies have yet to be carried out. Although it may be of interest to identify the disparate protein profiles associated with different histological subtypes of RCC for diagnostic information, identifying shared cellular pathways could potentially open the door for therapeutic approaches that can be utilized for patients with different RCC etiologies. Additionally, as more and more large-scale studies are performed for distinct cancer types, developing methodologies and bioinformatics approaches that allow for cross-platform comparisons would begin to link the common molecular features of histologically and anatomically distinct cancers. From these results, we can begin to use rationalized, precision-based therapies currently approved for one cancer type as viable therapeutic options in other cancer types. Several reports have already attempted this undertaking using publicly available datasets, including exploration of TCGA somatic, transcriptomics, and RPPA data [113], CPTAC gene expression data [114], and peptidome profiles [115], however, the results of these pan-cancer efforts are still preliminary. One approach that may find more utility as larger and larger sample sets are being investigated is data-independent data acquisition (DIA), which achieves similar, if not more, depth of proteomic characterization, with the added benefits of robust sample-to-sample quantitation and reduced instrument time needed for analysis [116]. Previous reports have used DIA to characterize tissues, serum, and urine from RCC patients [117–120], revealing similar profiles of dysregulated protein and cellular pathways expression compared to more traditional proteomic data acquisition approaches (i.e. data-independent data acquisition).

**Table 2:** Examples of proteomic approaches for characterizing RCC tissue, blood-associated (plasma/serum), or urine specimens

Biological source	# of samples	Experimental approach	# of differentially expressed targets	Citing report
Tissue	50	Used LFQ approach to identify proteins associated with tumor grade, profiling NAT and ccRCC tissues with Furman grades between 1 and 4	105	[42]
Tissue	75	Employed MALDI-MSI to identify differential expressed proteins associated with the tumor, tumor margin, and NAT regions	12	[56]
Tissue	194	Utilized proteogenomic approach; TMT-based quantitation for delineating differential global protein and phosphopeptide/phosphosite profiles between tumors and NATs	820	[20]
Serum	162	Profiled the serum peptidome in healthy controls, ccRCC patients, and ccRCC patients before and after surgical resection	18	[86]
Serum	99	Examined urine proteome profiles to discriminate ccRCC from healthy controls, benign kidney masses, and non-ccRCC urological tumors	27	[80]
Urine	254	Examined serum peptidome profiles to discriminate ccRCC from healthy controls, prioritizing discriminatory clinicopathological-associated features (stage, grade, tumor size)	15	[101]
Urine	90	Used LFQ to characterize the urinary proteome of ccRCC patients and healthy controls; stratifying ccRCC patients into good or poor prognosis groups based on Furhman grading	49	[92]
Tissue/EVs	40	Used an ex vivo model to profile extracellular vesicles (EVs) derived from ccRCC tumors and NATs	397	[112]

Another area in RCC biology that has yet to be fully examined at the protein level is an assessment of intratumor heterogeneity (ITH). It is well-established that individual tumors are heterogeneous, and previous reports characterizing ccRCC biopsies obtained from the same tumor have revealed genomic alterations thought to be mutually exclusive occurring in subclones in distinct anatomical regions of the tumor [121, 122]. To date, there have been no large scale proteomic assessments of ITH in RCC, thus information related to how individual subclones within the same tumor may influence protein expression is currently unknown. Determination of the heterogeneity of genetic, and subsequent proteomic profiles would aid in our understanding as to why patients develop disease recurrence or resistance to therapeutic intervention, as well as the selective intratumor/microenvironment influences that result in altered protein expression. Moreover, delineation of the degree of protein-level heterogeneity would confirm the validity of using tissue-based biopsies in the context of patient stratification for prognostic information and selection of targeted therapies. IHC is routinely utilized to visualize the abundance and spatial distribution of proteins, however, this technique does not allow for a full characterization of the proteome in a single analysis. As noted previously, tissue biopsies are routinely used for histopathological examination, and these sample sources may also prove useful for proteomic characterization. A more precise technique of tissue sampling is laser capture microdissection (LCM), which enables the isolation and separation of distinct cell types (e.g. epithelial, fibroblasts, immune cells) to reduce the degree of heterogeneity associated with resected tumors [123, 124]. Several “proof of concept” reports have shown the feasibility of pairing LCM and proteomic profiling to gain insight into the biological variations of distinct cell populations, albeit requiring the pooling of multiple samples to obtain sufficient material for analysis [125, 126]. Potentially illustrated in these studies is the major hurdle in ITH-based proteome characterization, specifically the limited amount of material available from individual tumors available for proteomic analysis. As genomic- and transcriptomic-based single cell analyses become more widespread [127], complementary mass spectrometry-based proteomic approaches that enable a relatively deep proteomic characterization of tissues using minimal sample input or single cells will be further developed [128–131], and will be applicable to explore this area of RCC biology.

## Conclusions

Proteomic technologies offer a comprehensive method for characterizing the functional biomolecules that regulate cellular processes, and determination of the aberrant protein expression patterns that are impacted by the disease state. The multitude of studies highlighted in this review are representative of the myriad of proteomic approaches that have been developed and leveraged to gain insight and a deeper understanding of RCC, and reflect the advancement in the field in terms of sample preparation strategies, instrumentation, and integration with other data types. Although there are several unmet clinical related to RCC, including discriminating various subtypes at the protein-level and an assessment of RCC intratumor protein expression, proteomics will continue to offer a complementary, yet robust technology for disease characterization.

## Data Availability

Not applicable.

## Abbreviations

2-DE: Two-dimensional electrophoresis
2DICAL: 2-Dimensional Image Converted Analysis of Liquid chromatography mass spectrometry
AQUA: Automated Quantitative Analysis
ccRCC: Clear cell renal cell carcinoma
chRCC: Chromophobe renal cell carcinoma
CPTAC: Clinical Proteomics Tumor Analysis Consortium
EVs: Extracellular vesicles
FFPE: Formalin-fixed paraffin embedded
IHC: Immunohistochemistry
ITH: Intratumor heterogeneity
iTRAQ: Isobaric tags for relative and absolute quantitation
LFQ: Label-free quantitation
NGS: Next generation sequencing
MSI: Mass spectrometry imaging
MudPIT: Multidimensional Protein Identification Technology
NAT: Normal adjacent tissue
PCA: Principle components analysis
pRCC: Papillary renal cell carcinoma
PTM: Post-translational modification
RCC: Renal cell carcinoma
SRM: Selective reaction monitoring
TCC: Transitional cell carcinoma
TCGA: The Cancer Genome Atlas
TMA: Tissue microarray
TMT: Tandem-mass-tag
VHLD: VHL disease
